# The affordances of art for making technologies

**DOI:** 10.1177/10597123221132898

**Published:** 2022-10-18

**Authors:** Erik Rietveld

**Affiliations:** University of Amsterdam, 1234 Amsterdam UMC Location AMC, Psychiatry, Amsterdam, Netherlands

**Keywords:** Affordances, visual art, making, humane technologies, material playgrounds, Skilled Intentionality Framework

## Abstract

With this inaugural lecture as Socrates Professor on the topic of Making Humane Technologies, I aim to show that artistic practices afford embedding technologies better in society. Analyzing artworks made at RAAAF, an art collective that makes visual art and experimental architecture, I will describe three aspects of making practices that may contribute to improving the embedding of technology in society: (1) the skill of working with layers of meaning; (2) the skill of creating material playgrounds that afford free exploration of the potential of new technologies and artistic experiments; and (3) the skill of openness to the possibility of having radically different socio-material practices. I will use images of several RAAAF projects to make these skills involved in making more tangible. It is artistic skills like these that can contribute to a better societal embedding of technologies.

## Introduction

This Socrates Chair is titled: Philosophical reflection on making and societal embedding of technologies in the humanist tradition. To understand what I mean by the societal *embedding* of technology, imagine a city that has embraced cars that run on solar energy.^1^ This looks like potentially important progress on the issue of sustainability. If, however, the need to charge the solar panels leads to many trees being cut so that the cars get enough sun and can charge better, this promising new technology will turn out not to be very well attuned to the rest of society. Something that people—and many non-human animals—value in their living environment has been neglected: the trees. The makers of the solar car seem to have ignored that trees are meaningful in people’s lives. This solar car is a case of high-tech without human touch. To make humane technologies, one needs to pay attention to how the technologies are taken up in the life-world of people, in all their variety, and how that intervention in their lives feels for these people.^2^ Perhaps the solar car’s makers were too concerned with solving the technical problems to consider what it would mean to live well with solar cars in real life. In any case, the new technology is not, or at least not yet, well embedded in the shared living environment.

An important question that I would like to work on in the coming years and that I would like to share with you today, is what could visual art afford for people involved in making technologies? Could artistic practices show us ways to embed technologies better in society?

Visual art is of course a very broad notion. To make it more specific, I will today be focusing on the way we make it in our own practice, at RAAAF [Rietveld Architecture-Art-Affordances]. RAAAF is a multidisciplinary studio, operating at the cross-roads of visual art, architecture and philosophy. It was founded in 2006 by my brother Ronald Rietveld and me. At RAAAF, we make seemingly impossible site-specific interventions in the living environment based on an urge to explore and reflect on what is possible in contemporary life.

**Figure 1.**
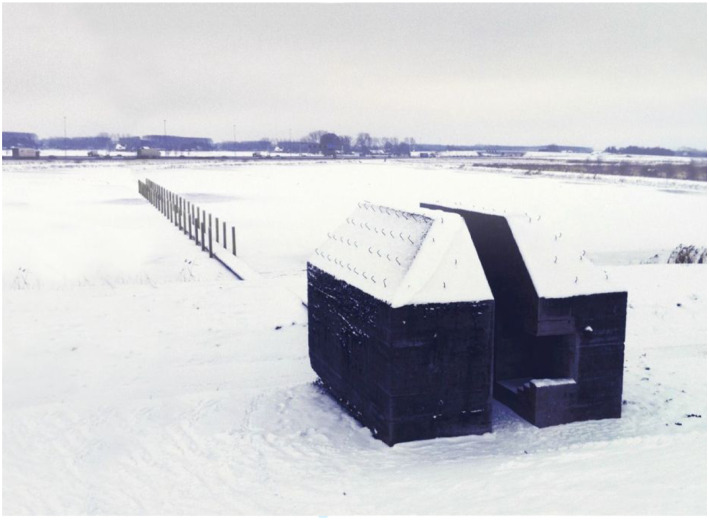
Bunker 599, 2010, RAAAF | Atelier de Lyon. Photo: Allard Bovenberg.

Here is an example of the kind of visual art that we make (Figure 1). This artwork is titled *Bunker 599*. We made it in collaboration with Atelier de Lyon. This bunker is a municipal monument that we cut through. After this intervention, the bunker became a national monument. The sliced bunker is also part of the UNESCO World Heritage listed defense line, the New Dutch Waterline.

**Figure 2.**
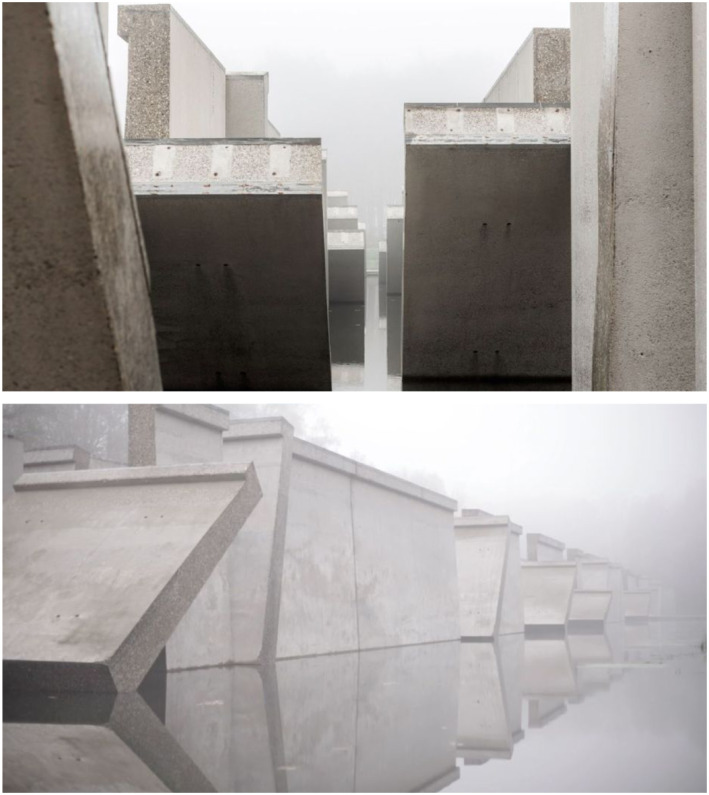
Movie stills; the making of Deltawerk// Link to movie: The making of Deltawerk//.

To further clarify the kind of visual art that I will be focusing on today, I would like to show you a short movie. *The making of Deltawerk // *shows another of our works made in collaboration with Atelier de Lyon, just like the *Bunker 599*. *Deltawerk // *is a 250 meter long artwork at the Waterloopbos, a site where 75 engineers have been working on all kinds of tests and experiments for the construction of the Delta Works that aimed to protect The Netherlands from flooding (Figure 2).

**Figure 3.**
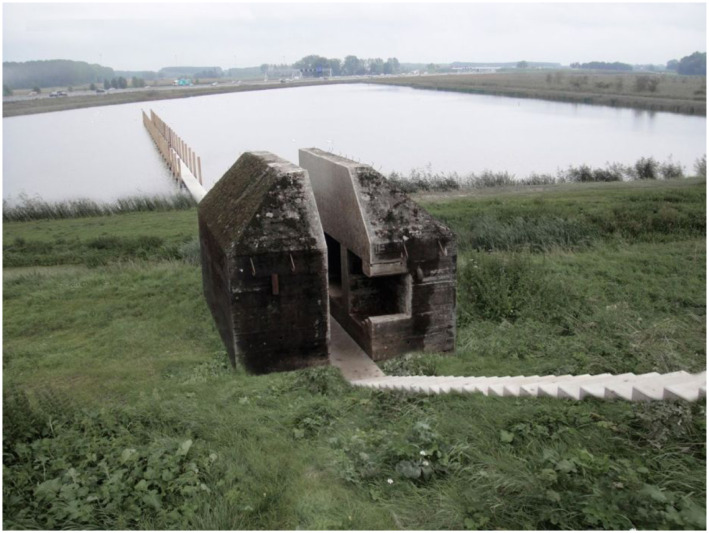
*Bunker 599*, 2010, RAAAF | Atelier de Lyon. Photo: Allard Bovenberg.

With *Bunker 599* (Figure 3) and this movie in mind let us turn now to the question that will occupy me in this lecture: what can visual art offer to people making technologies? A first clue is that one of the things that many artists are really good at is *opening up radically new perspectives on what is possible and meaningful in human life*. Ways of working in the arts, artistic practices, can lead us to question and investigate what we ordinarily take for granted; and help to imagine how we could do things differently—live differently.^3^

The take home message of my lecture today is that artistic practices afford embedding technologies better in society. I will describe three aspects of our making practices at RAAAF that may contribute to improving the embedding of technology in society: the skills of working with layers of meaning; the creation of material playgrounds; and the openness to the possibility of having radically different practices.

1. Working with layers of meaning: Our concern in making RAAAF's site-specific installations is not with solving problems, or with the design of instrumental objects, but with working with the layers of meaning our artworks can open-up.
2. The setting-up of material playgrounds: At RAAAF, we take pleasure in joining forces with materials, intuitively exploring what can be done with materials in our engagement with them (Malafouris, 2014). *Material playgrounds* afford free exploration of the potential of new technologies and artistic experiments.
3. Openness to the possibility of having radically different socio-material practices: The process of making of our interventions is characterized by an important openness to unconventional possibilities for living in ways that are very different from what one generally tends to take for granted. We enjoy imagining and creating such new worlds. Often artists go further or go beyond where anyone has ever been so far.

I will use three RAAAF projects that illustrate each of these three aspects of making artworks. This will foreground some of the *skills* involved in making our interventions. It is these skills that might contribute to better embedding of technologies.

**Figure 4.**
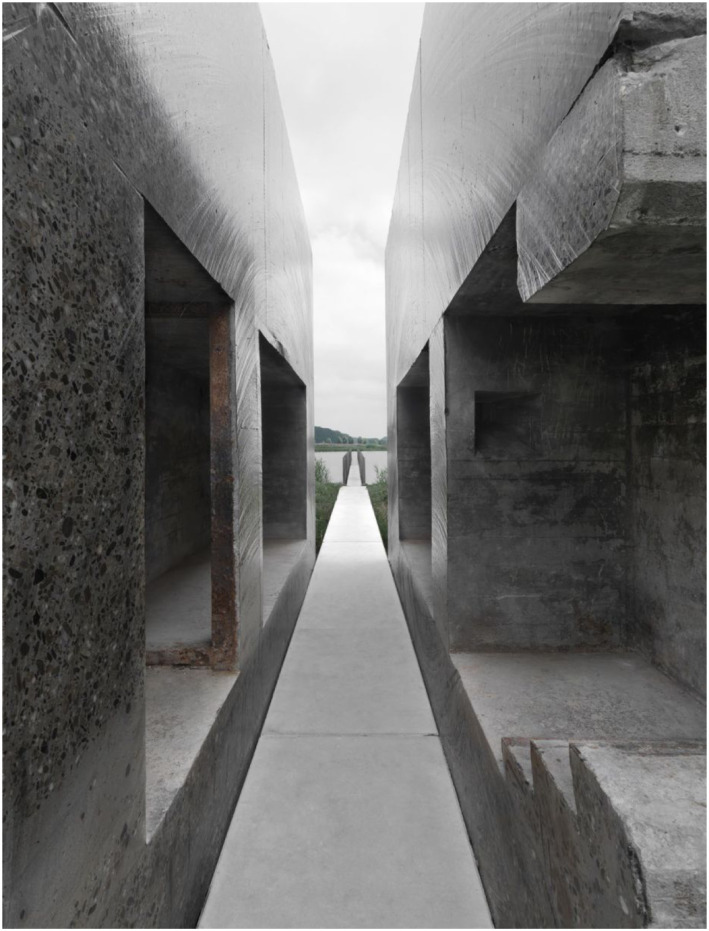
Inside the sliced object. *Bunker 599*, 2010. RAAAF | Atelier de Lyon. Photo: Allard Bovenberg.

## Section 1. Working with layers of meaning

**Figure 5.**
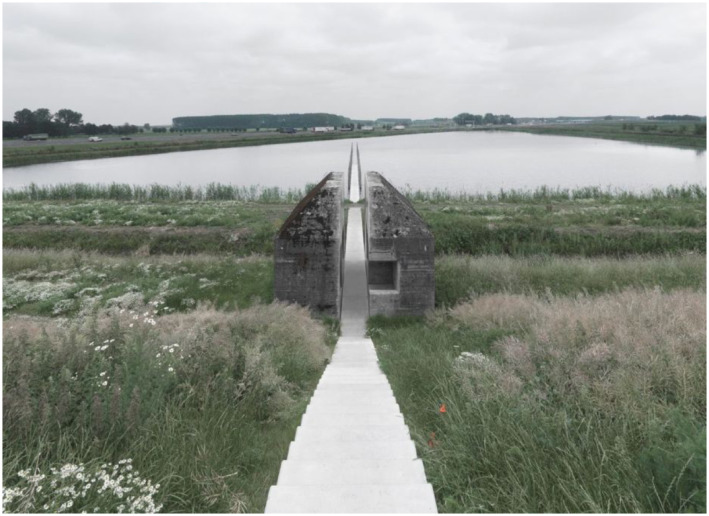
*Bunker 599*, 2010, RAAAF | Atelier de Lyon. Photo: Allard Bovenberg.

The first skill is for opening-up and connecting layers of meaning in the process of making a site-specific artwork. I will take *Bunker 599* as my example, which I already briefly introduced above. You can imagine that someone is walking along the dike and encounters the bunker, being surprised and full of wonder at what is happening there, looking carefully if she sees it well. The person goes down the sloping dike path and explores the inside of the cut object, encountering for the first time a new perspective on seemingly indestructible bunkers (Figure 4). The material structure of it becomes clear: the several meter’s thick concrete “roof” and the reinforcement steel that is inside such a bunker’s concrete are made visible. The sliced object affords novel possibilities for exploration: for example, looking closely at the beautiful structure of the cut concrete, imagining where people were standing in times of war or the fear of a soldier inside. It also opens up a perspective on how the Dutch military were conceptualizing defense strategies in 1940. The Dutch were trying to strengthen their defense against Nazi Germany by making a series of this type of bunker. The artwork renders this history visible and tangible, that is, *experiential*. Sedimented layers of meanings are brought to the surface as it were, relating to the bunker and its materiality; its location in the New Dutch Waterline; the water that was used as part of the Line’s defense system; and so on (Figure 5). The artwork evokes affective experiences in people engaging with it. People with different interests will have different experiences of the meaning of this artwork.

For us as makers it was important that the bunker should offer multiple layers of meaning. The primary act by RAAAF | Atelier de Lyon was not adding something, it was cutting a seemingly indestructible object; slicing the bunker open. By taking away something rather than adding, the bunker’s embedding in multiple practices is opened up to experience.

**Figure 6.**
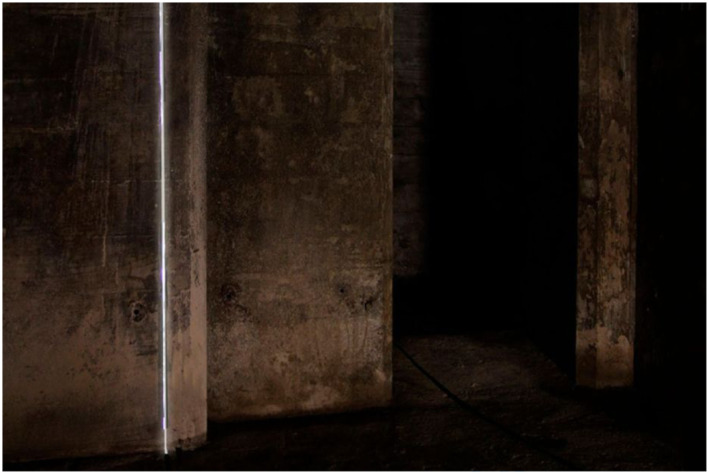
First cut. *Bunker 599*, 2010, RAAAF | Atelier de Lyon. Photo: Allard Bovenberg.

As mentioned, it was a municipal UNESCO nominated monument when we cut into it (Figure 6). The bunker’s status as cultural heritage is part of the context that generates the meaning of our intervention. The standard practice is to consider monuments as objects with a commemorative value that need to be protected and persevered. Cutting into and opening up such an object seems to contradict, and therefore question, this convention.

By compromising the physical integrity of this historical object, the artwork questions our understanding of what monuments are.

Now let me briefly reflect philosophically on the relation between conventions or established practices and meaning. We can think of meaning as having different sources (Merleau-Ponty, 1945/2002, Merleau-Ponty, 1968/2003; Wittgenstein, 1953; Rietveld, 2008). Some of the meanings attaching to aspects of the environment (including artworks) come from *the person’s embodiment*: from the caring and selective sensitivity to the environment we get through the way our body is made up. It is on the basis of an individual’s embodiment, which has been structured through a history of engagements with the world, that things can speak to them; move them to act. What *matters* to the person will make all the difference for how they will experience something that they encounter, including artworks. But, crucially, also what the person *can do* makes all the difference: the skills, abilities, and habits that one has. If you can understand English well these words will have a deeper meaning for you than if you just started learning English.

Much of the meaning people find in the world originates in *socio-material practices* in which people partake. Practices are relatively stable patterns of behavior manifested over time by a multiplicity of people. Human practices are both social and material, or socio-material (Mol, 2002). *Bunker 599* is situated in practices of heritage conservation and monumental policies, practices of visual art, practices of military defense, including the New Dutch Waterline as a defense system, and the practice of English language use. But also an everyday practice, like walking for relaxation say, can contribute to the layers of meaning of this artwork.

One thing that is both interesting and somewhat disturbing about practices is that even though they are a source of meaning, we often take them for granted. We take for granted that we preserve monuments, that we put objects of cultural heritage on a pedestal in a museum, that we do not touch them, and avoid that anyone destroys them. An intervention like *Bunker 599* changes this and makes tangible that heritage practices could be very different.

The notion of *affordances* (Gibson, 1979/1986) in the title of my lecture ties together socio-material practices and our embodied abilities. The term “affordance” means a possibility for action that the socio-material environment offers to us. You should understand action here in the broadest sense of things people are able to do. The term “affordance” as I use it, is thus a very rich notion because there are many different kinds of actions and thus a large variety of action possibilities (Rietveld & Kiverstein, 2014). Action includes walking and sitting, but also preserving monuments, and quite crucially, reflecting on artworks and, say, imagining what it was like to be inside the bunker in spring 1940. Reflecting and imagining are also actions people can do skillfully.

There are close ties between affordances, practices, and skills (Rietveld, 2008; Rietveld & Kiverstein, 2014; Rietveld & Brouwers, 2016; Van Dijk & Rietveld, 2017; 2018; Rietveld et al., 2018; Kiverstein et al., 2019; Zijlmans, 2018). Practices educate and entrain their participants and provide a common ground. Practices can be seen as a particular communal way or pattern of engaging with certain affordances rather than others. The education of attention of novices by more experienced practitioners makes it possible for people to acquire skills. This process of learning changes the person’s embodiment and affective sensitivity to the environment (Colombetti, 2014). It makes it the case that for those who partake in the practice some affordances have more significance or invitingness than others (Rietveld, 2008, p.992). A side-effect of this skilled bias or selectivity is that some affordances tend to be ignored by people in the given practice. Practices typically generate a selective openness to those affordances that allow us to go on in the same way as the other practitioners (Wittgenstein, 1953), to act according to the established norms, but the cost of that conventional selective openness is that people also habitually ignore many of the more unorthodox affordances.

Artworks generate meaning by offering new affordances, new possibilities for engagement with the world, including affordances for reflecting on the meaning of the artwork. Artworks offer possibilities for reflection both to their makers and to other people experiencing them. What takes form in artistic practices of playing with materials and in the artistic process of making more generally are often *unconventional* affordances. For example, slicing open the seemingly indestructible bunker was an unconventional action possibility that was realized by RAAAF | Atelier de Lyon. Such a provocative intervention has the power to disturb our habitual routines, like passing by the bunkers without noticing them during a daily recreational walk, or treating them as monuments to be conserved and remain untouched. By breaking this routine, the intervention makes that many of the bunkers of the New Dutch Waterline are suddenly triggering our imagination again and that these monuments do not fade from public imagination and memory (Rietveld et al., 2017; Rietveld & Rietveld, 2017).

Making an artwork is working with layers of meaning. Crucially, in the process of making at RAAAF, we are typically very sensitive to how our interventions will influence and intervene in different practices. That kind of awareness is important because the meaning we make with our work is in part derived from these practices. So, when we make something at RAAAF we attune to, that is, coordinate with, these practices (Van Dijk & Rietveld, 2018). We are very *sensitive to how what we will be doing relates to some of these different practices*, say for instance practices of monument conservation, or of people encountering a bunker when going for a walk. There are of course limits to what one can anticipate as maker.

The realized artwork has an openness or communicative power that goes beyond what we as makers were aware of in the process of making. It can affect people in unexpected ways. Someone from China experienced the cut through military object as a moving object of peace, for example.^4^

Of course, some artists are less reflective, dealing with their materials mainly intuitively, but then often they will team up with a curator who does the work of situating the artwork; placing the artworks in the context of wider practices. This kind of collaboration between artist and curator is so common precisely because art offers the possibility to work with layers of meaning and open up new meanings.

I have now described the first aspect of what art can afford for makers of technologies: the layers of meaning that we attune to in the process of making and that are skillfully “woven into” the formation process of the artwork (cf. Ingold, 2013). Artists master the valuable skill of relating deeply to the different practices that are the sources of meaning of what they make.

## Section 2. Material playgrounds

**Figure 7.**
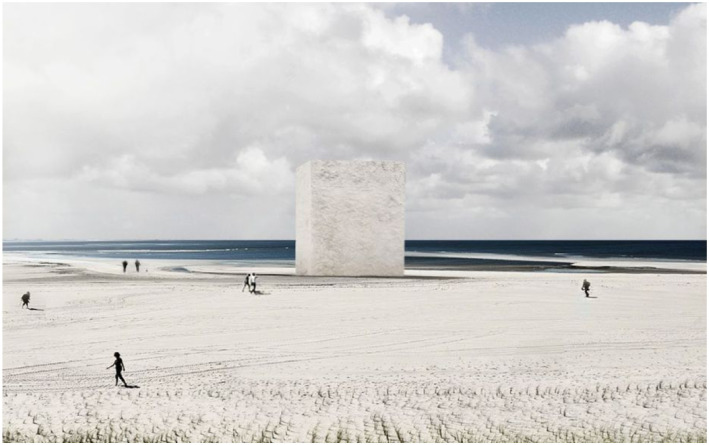
*Sandblock*, 2019, RAAAF | Atelier de Lyon.

**Figure 8.**
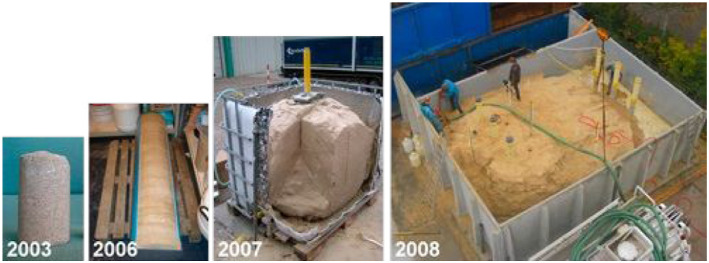
Scaling up from 10 cm to 100 m³ biological sandstone. Photos: Deltares, TU Delft & Volker Wessels.

The second skill I will describe is the exploration of the possibilities materials offer through the creation of material playgrounds. Here is an example. This is a 12 meter high sandblock on the beach that RAAAF and Atelier de Lyon are currently proposing as an artwork here in the Netherlands (Figure 7). *Sandblock* (working title) will be made by bacteria. By joining forces with bacteria, we can transform sand into hard bio-sandstone to realize this massive sculpture on the beach. In Figure 8, you can see the development of this technology. In 2003, it was possible to make in the lab at TU Delft a small (10 cm) piece of this material using the bacteria to generate the biological sandstone. The technique has evolved over the years and was in 2008 scaled up in the lab to 100 cubic meters thanks to fundamental research (see Van Paassen et al., 2010). Until now, the engineers at TU Delft were able to make a biological sandstone structure measuring about 8.0 by 5.6 by 2.5 m.

The application of this fascinating technology has, however, ended up in the “valley of death” where many innovations get stuck because there are no prospects for them to be taken up in a practice. One of the possibilities, a visual arts project can offer to makers of new technologies is a material playground for scaling up technologies like bio-sandstone. The size of our sand block, which is several times higher than what the engineers have been able to make so far, will challenge them to scale-up but also give them the kind of free space for exploring the possibilities for scaling up.

But importantly, embarking on the project of creating the artwork also amounts to the creation of an explorative journey for ourselves at RAAAF: the creation of our own material playground. Experimenting and freely tinkering on scale 1:1 with new technologies and materials, we can imagine new futures. At the same time, the *Sandblock* project helps us at RAAAF to scaffold the public’s imagination. The experience of the 12 m high structure of sand that only recently was lying loose on the beach, creates new spaces for the mind. It enables people to imagine new sand worlds grown from the local materials, for example.

We have been fascinated by the bio-sandstone technology for years. Even when the studio started, back in 2006, we were already thinking of possibilities of this bio-sandstone for creating all sorts of interventions. In general, material playgrounds at RAAAF allow for freely and intuitively *exploring experientially* what the affordances of a material experiment or a given technology are. This starts out of a passion for making without having to worry about any instrumentalization or future use. Often, we just start a process of material engagement without knowing where we will end up. This kind of fascination-driven playful exploration can lead to the discovery of radical possibilities and meanings that the engineers involved had never considered, and sometimes to new ways of living with the technology.

**Figure 9.**
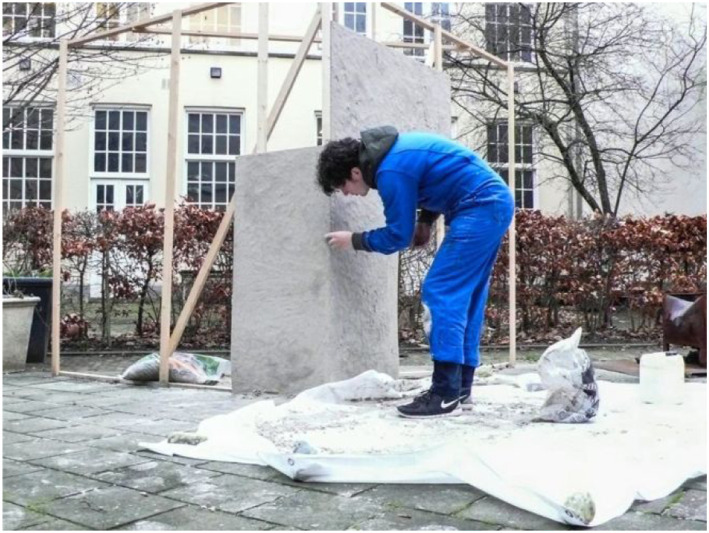
Material exploration for Sandblock. Photo: Ricky Rijkenberg

**Figure 10.**
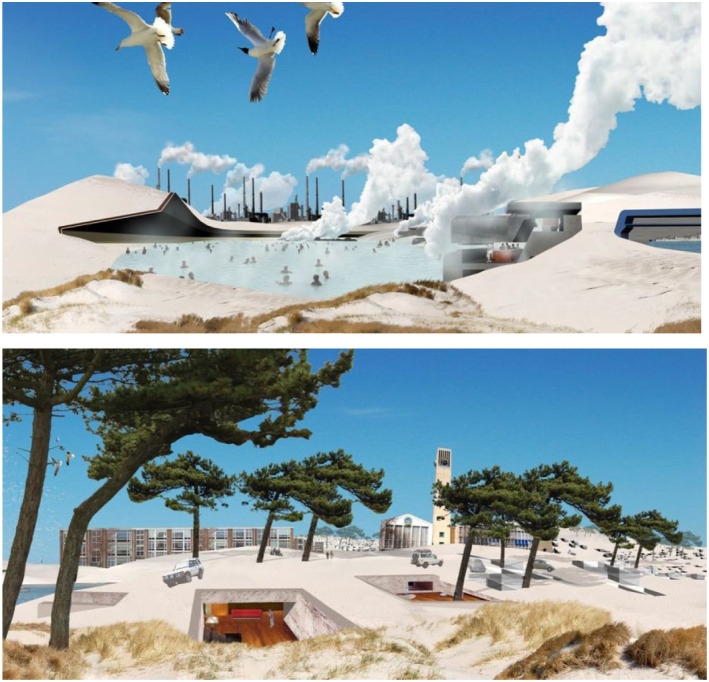
Prix de Rome architecture 2006, first prize Ronald Rietveld. Generating *Dune Scapes*, RAAAF.

For making *Sandblock*, we will have to really stretch what is technically possible (Figure 8). One of the material playgrounds we recently made at RAAAF for exploring its aesthetic potential you can see in Figure 9. However, the first moment that the technology showed up at the studio was in the process of working on the winning Prix de Rome project that my brother Ronald made in 2006. In that project, titled *Generating Dune Scapes*, you see in the lower half of the Figure 10 a part where sand was transformed into biological sandstone near the Kennemer Dunes and IJmuiden beach. At that time, the plan was to inject the bacteria in the sand and create four walls, then take out the sand in between the walls (Figure 10).

Once you realize that the billions of new people who will be living on our planet in 2060 will need houses, one of the urgent challenges for architecture is to provide places for them to live. Places that are built in a sustainable way and use bio-cement and locally available material, like sand from deserts and river banks, rather than bricks or concrete. From the perspective of global warming, this is an urgent challenge because cement is the key ingredient of concrete buildings, and the cement industry already had one of the largest CO2 footprints globally in 2016.^5^ And, ideally, architects would provide houses that do not need air conditioning but are naturally fresh, for example, because they are underground.

**Figure 11.**
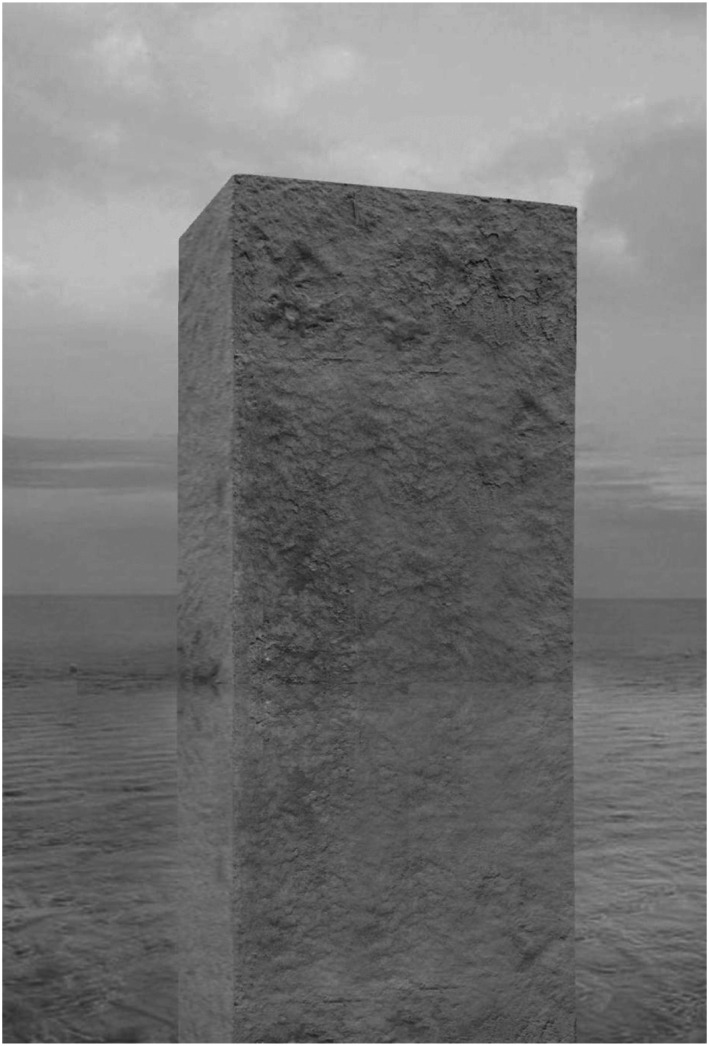
*Sandblock*, 2019. RAAAF | Atelier de Lyon.

We have been intrigued by this new material for many years and what we would like to do next is to join forces with the engineers at TU Delft and their bacteria (see Van Paassen et al., 2010), and together stimulate reflection on this new era for architecture. The project we envisage will mark this new era not by means of a house or any other practice application, but by making an artwork in the form of the huge *Sandblock*. Its aesthetics will afford people to build affective relations to the object and the new material (Figure 11). We will make the work not for some practical application but out of our own fascination, pleasure, and interests in experimenting and playing with bio-cement and bio-sandstone. We will use locally available sand and transform it on site with the help of bacteria. Our art project also provides a unique opportunity for the makers of this technology to experiment, making available to them a material playground to scale up in real life, and hopefully to get their innovation out of the valley of death.

This was the second aspect that artistic practices have to offer to makers of technologies for embedding these technologies. The first skill I mentioned was that of working with layers of meaning by being sensitive to multiple meaningful practices. The second skill was the creation of material playgrounds for exploring experientially the potential of the things we make.

## Section 3. Openness to the possibility of having radically different practices

**Figure 12.**
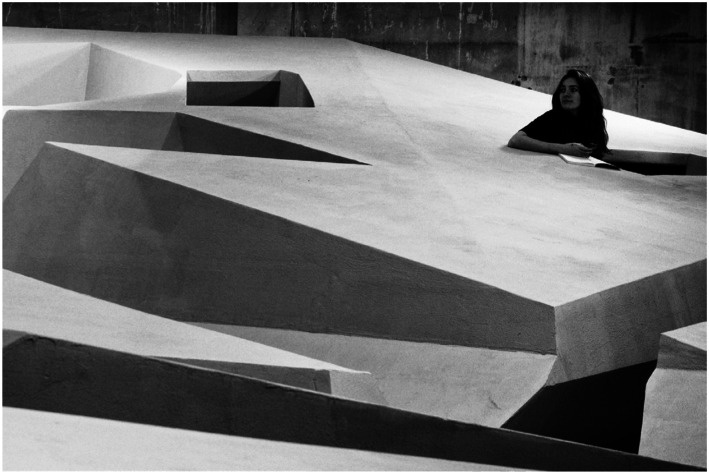
The End of Sitting, 2014, RAAAF | Barbara Visser. Photo: Ricky Rijkenberg.

The third skill I will discuss is that of being open to possibilities of having radically different practices or ways of living. Importantly, the openness to unconventional possibilities is *not something that is just happening in the head* of the artist; it is a relational phenomenon, and so something that we can also materially scaffold. In a project titled *The End of Sitting*, we were interested in exploring what a world without chairs and without sitting could look like (Figure 12). Can we imagine a different world where supported standing would be the norm? What would living in such an environment be like?

To explore these questions, we started to play with the available materials in the studio of Barbara Visser, the visual artist with whom we collaborated. In this material playground, we began simply by experientially exploring the possibilities for working in different positions.

**Figure 13.**
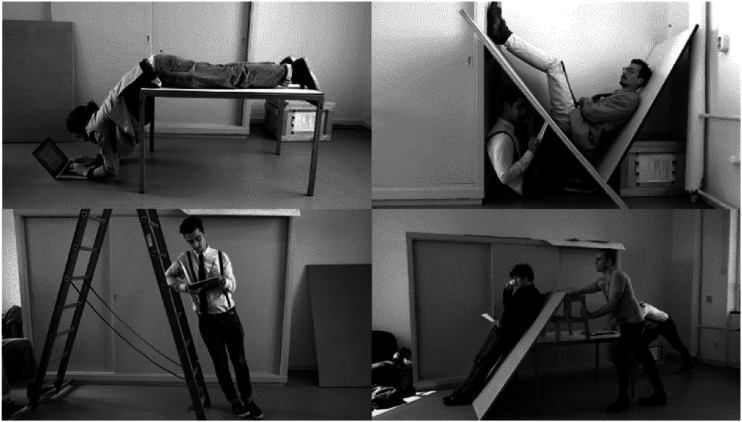
Playful exploration at RAAAF. Photos: Barbara Visser.

**Figure 14.**
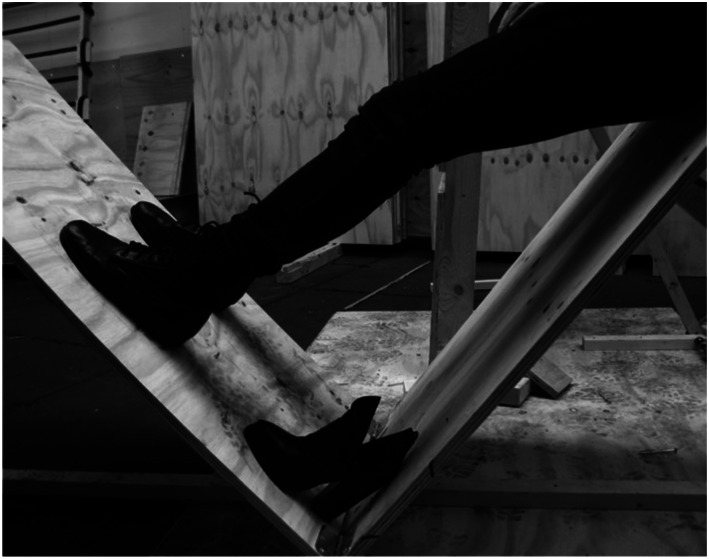
Tilted feet. Photo: RAAAF.

Here you see some examples of the playful exploration of the human landscape of affordances. In that process, we made many discoveries (Figure 13). For instance, we discovered that a certain angle feels great for reading when you are standing in a supported position. When you are leaning back and your feet are elevated, it feels even better (Figure 14). When you place a laptop on a support in front of you while you are supported standing, it also feels better and often you even forget that you are working standing. We were playing with the body in interaction with materials, improvising and exploring what kind of affordances for supported standing we enjoyed. The body of the person makes all the difference for how they experience these real-life mock-ups.

**Figure 15.**
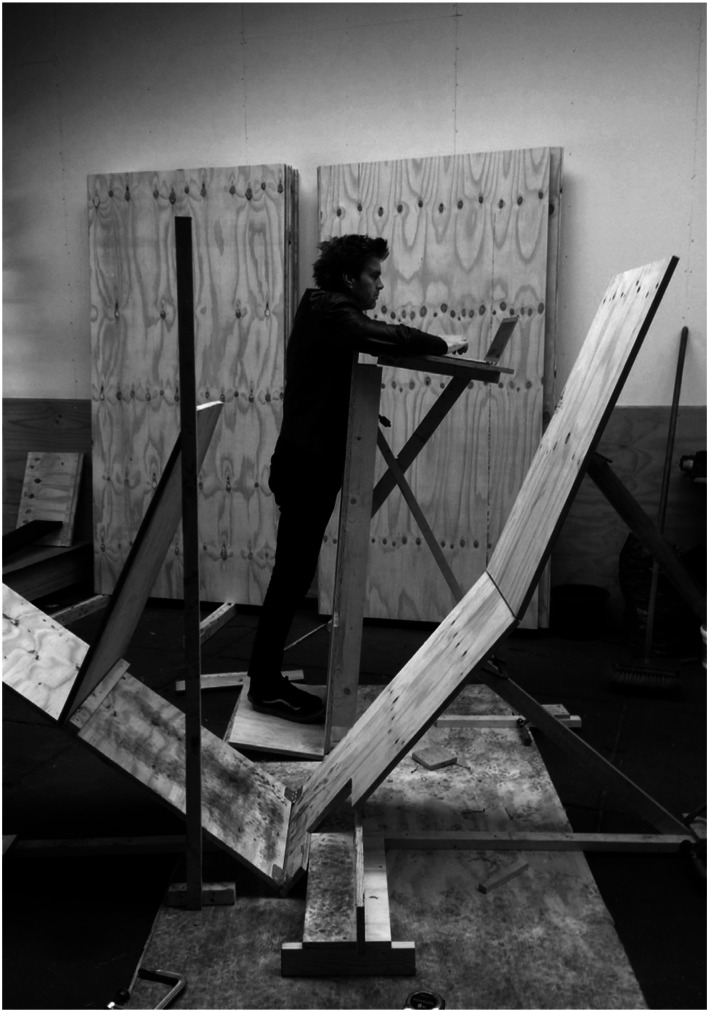
A position that demanded adjustment. Photo: RAAAF.

In Figure 15, you see changeable scaffolds for supported standing. Everything can be adjusted and one of the main things we would do in the process was feeling what we would experience as good and what felt wrong or awkward. Affective experiences like these give direction to the process of experimentation and improvement in making (see Rietveld, 2008; Van Dijk & Rietveld, 2018). The position I am standing in Figure 15 was a position that did not feel right and demanded adjustment.

**Figure 16.**
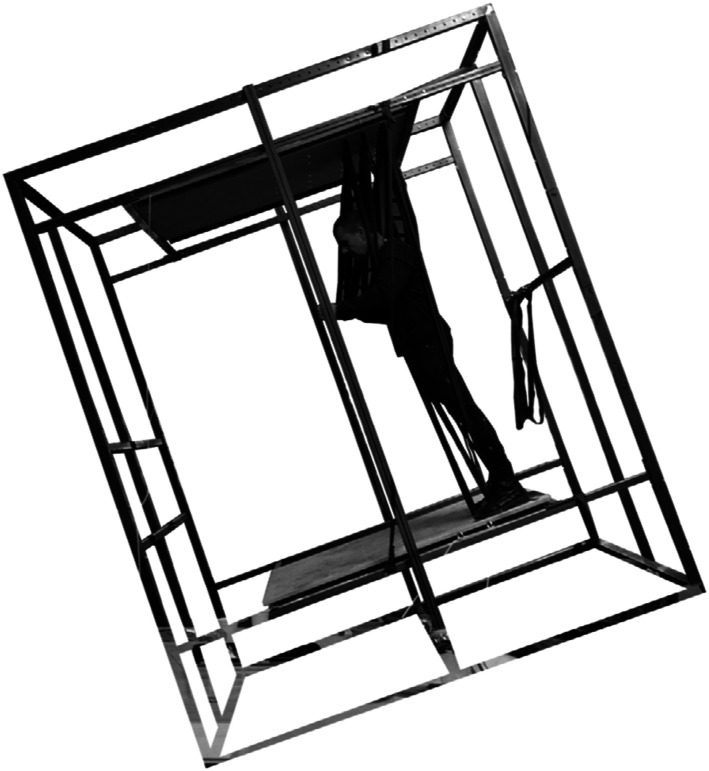
Tilted frame. RAAAF | Barbara Visser.

**Figure 17.**
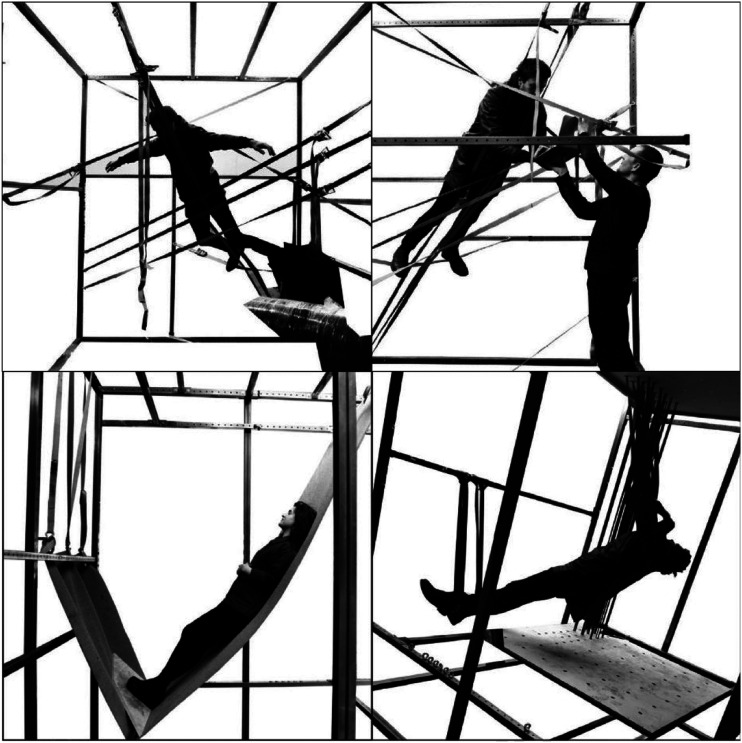
Material playground: explorations in frame. RAAAF | Barbara Visser.

**Figure 18.**
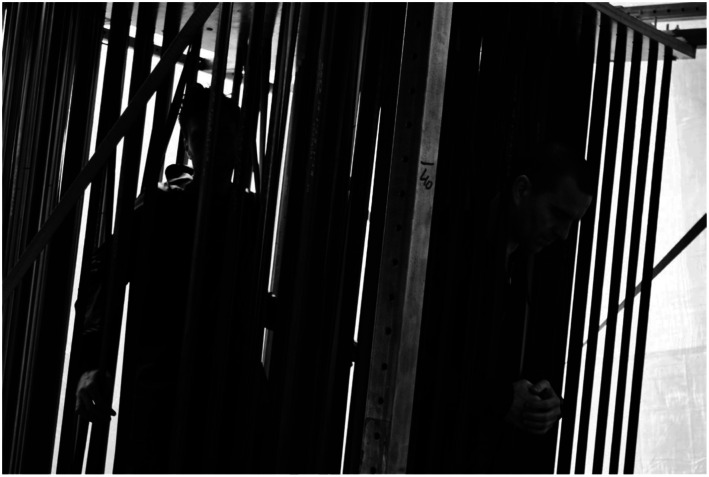
Feeling the position. RAAAF | Barbara Visser.

To further support our own process of coming up with new ideas we built a strong metal frame in which we could test out all kinds of materials. Through bodily and affective engagement with the materials suspended in the frame we could explore what we enjoyed and what positions did not feel good. As you can see (Figure 16) the frame that we made, could be tilted so that we could enjoy leaning back and having sloped feet support. We suspended many different kinds of materials: rubber inner tires of bikes, ratchet straps, carpet, rubber sheets, wooden planks, etc. Figures 17 and 18 show some more bodily explorations of what is possible with the materials.

This process of experimentation for *The End of Sitting* is thus another example of what I mean by creating a material playground. It helps us at RAAAF in opening up in an experiential way to unexplored or unconventional possibilities. A material playground is also a shared structure or set that supports us jointly as a team with understanding experientially the potential and meaning of what we are making.

**Figure 19.**
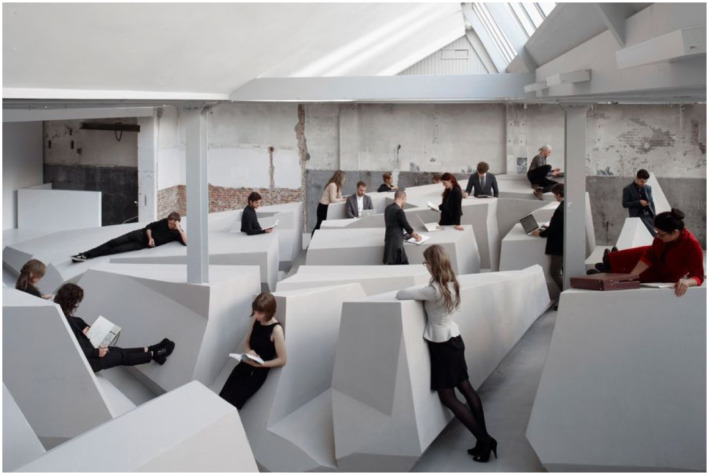
The End of Sitting, RAAAF | Barbara Visser, 2014. Photo: Jan Kempenaers

**Figure 20.**
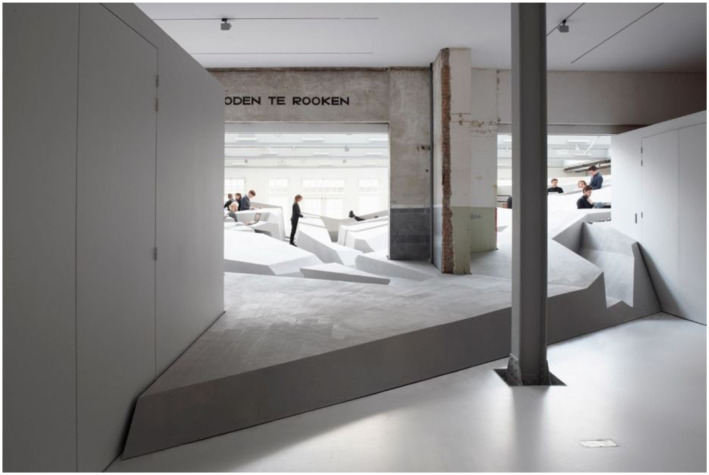
The artwork at looiersgracht 60. Photo: Jan Kempenaers.

Some images of *The End of Sitting* art installation. The people here (Figure 19) are leaning back or supported standing, so even though it may seem that some are sitting, they are not, and are very much using their big leg muscles. The artwork (Figure 20) is both a site-specific installation and a local landscape of many affordances for leaning back, standing, hanging, and moving around. Importantly, what *The End of Sitting* installation invited visitors to do, was becoming aware of their habitual ways of living and what they take for granted. It confronted them, for example, with their normal sedentary lifestyle. Visitors might also suddenly come to realize the way in which the things they do are enabled by the affordances available in our ecological niche. In other words, the art installation afforded reflection.

Building the installation and seeing people enjoying the unconventional structure also creates hope: it suggests that it is possible to change entire practices; that we could live very differently. People could experience what it would be like to live by a different set of rules. So, the socio-material practices in which we are situated are *changeable* and this installation makes that very tangible. It lets you experience the changeability of the norms or practices that we take for granted.

The magazine *Wired* (Rhodes, 2014) called *The End of Sitting* “The Weirdest proposal for the Office of the Future,” and people from all over the world seemed to either love or hate the installation. It is interesting to note that *Wired*, a magazine for new technologies, took *The End of Sitting* to be a proposal for how to design the office of the future rather than as an artwork that questions the established practice.

You may wonder though what all this has to do with embedding technologies. To understand this, it is important to remember that technological innovation need not be digital, robotic, or nano. *The End of Sitting* installation was an innovative rethinking of both the practice of interior architecture and our contemporary “sitting society” more generally; it afforded to rethink our living environment starting from supported standing. Moreover, people working in Science and Technology Studies (e.g., Mol, 2002; Verbeek, 2011; Aydin et al., 2018) would call the chairs that you are sitting on sitting technologies, and pen and paper writing technologies. So, the notion of technology is a very broad notion and I think it is important to avoid starting by assuming that everything that is interesting technology should be nano, digital or have sensors. Neither should innovation necessarily be high-tech. Crucially, for making humane technologies, that is, technologies that are well embedded in the human form of life, we should start from thinking of body subjects, in all their variety, engaging with the technology in their life world and how that feels; how they experience the world differently and relate to the socio-material practices they are situated in; and the different layers of meaning that a certain kind of technology can bring into the world.

The empirical scientists Rob Withagen and Simone Caljouw used the artwork as a living lab to investigate how it would be to work while supported standing (Withagen & Caljouw, 2016). The art installation raises all sorts of questions: How do people experience working in the installation as compared to a conventional workspace? What does it mean for their wellbeing? How much energy do people use? If you were to work eight hours a day in it, would you spend enough energy to replace the gym? These human movement scientists and ecological psychologists did a study with four different camera points to observe how their subjects behaved in this artwork and interviewed them. *The End of Sitting Cut Out* travelled to the University Medical Centers of Groningen, Amsterdam, and Maastricht. During a workshop at the Amsterdam University Medical Centre ergonomists were invited to explore it by epidemiologists. For another study, researchers observed how people spontaneously engage with the installation in a public space at the university (Renaud et al., 2017). In short, this artwork afforded all sorts of scientific explorations because it was radically unconventional. *The End of Sitting* went where no one had gone before and that is one of the things artists are really good at: exploring new territories and possibilities.

**Figure 21.**
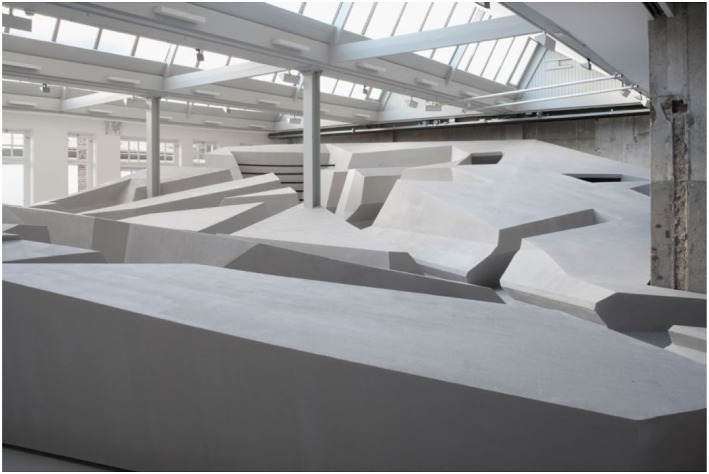
A materialized philosophical worldview. *The End of Sitting*.

**Figure 22.**
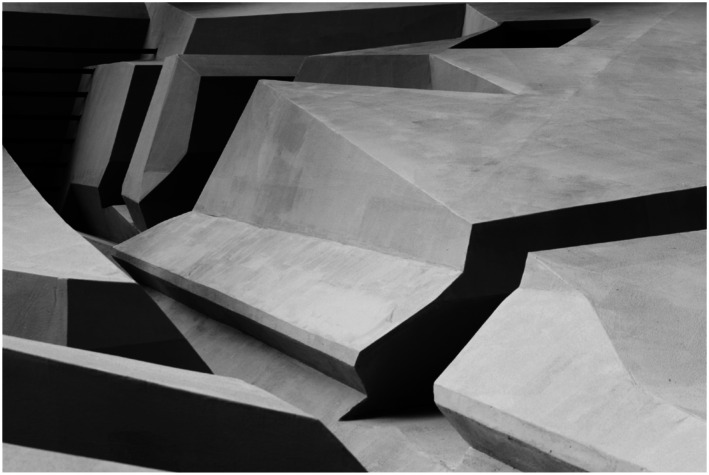
Close up of *The End of Sitting*. Photo: Ricky Rijkenberg.

In sum, what *The End of Sitting* project did for both its makers and the public was opening up unconventional possibilities. The process of making it involved the creation of a material playground for experiential exploration of the possibilities of technologies for supported standing. The site-specific installation explores what it would be like to live in a world without chairs, where standing would be the new norm. Interestingly, this “weird” artwork offers a particular kind of affordance for so-called “higher” cognition: it invites reflection on our habitual sitting behavior and the sitting society in which we live, thus raising awareness of what we tend to take for granted. More importantly even, the installation foregrounds in an experiential way that our human engagements with the world are *structured by affordances* (Rietveld et al., 2018). As such, *The End of Sitting* (Figures 21 and 22) materializes a philosophical worldview (Rietveld, 2016). People are embodied minds situated in a rich landscape of affordances (Rietveld & Kiverstein, 2014). Although we currently take for granted that academic philosophers write texts, typically without images, *The End of Sitting* tries to imagine a practice in which academic philosophy is done also in a non-textual, visual, and tangible way (cf. Alva Noë, 2015).

The process of making this artwork illustrates the skill of being open to the possibility of having radically different practices, and of breaking our habits more generally, which is a very important affordance in our contemporary life. The various mock-ups and the art installation afford feeling, affectively experiencing, and imagining what it would be like to live by a different set of rules, to live the good life differently.

## Conclusion

In short, I propose that artistic practices afford embedding technologies better in society. My examples from the artistic practice of RAAAF offer suggestions of how this can be done: it is an integrated set of skills that allows for this. First, a crucial skill in the process of making is that of being sensitive to and working with the different layers of meaning that interventions can have for people. In order to make technologies that are well embedded in the human form of life, engineers could develop the skill of relating more sensitively to the socio-material practices they are intervening in. They could try to become aware of the different layers of meaning that a certain kind of technology could offer to different people in these different practices. Second, an important skill in the process of making is setting up material playgrounds for exploration in which embodied sensing, feeling, and the pleasure of making take the lead. Third, artists have the skill of opening up to possibilities for questioning and transforming established practices. They open up to unconventional affordances, including provocative possibilities for changing what we—and this “we” can include the artists themselves—take for granted; for breaking habits. They are also masters in making tangible that we could live by different rules. Researching this skill set and “translating” or connecting the results to situations in the practices of engineers and scientists will be a project in my Socrates chair.

One of the established practices that I feel an urge to question is that of doing academic philosophy. Normally, philosophers write texts without images. However, as Alva Noë (2015) has argued, artworks that question our conventional practices and norms can be seen as a way of doing philosophy. Can we further develop this “philosophy without text,” an interesting philosophy of “show, don’t tell”? Can academic philosophy be done non-discursively, by visual means? Can philosophers join forces with visual artists to investigate non-verbally how we could live differently and perhaps better? To explore and unlock its potential, I believe it is important for the practice of philosophy to develop the genre of philosophical art installations further in the future.

Before we turn to a final movie, I would like to say a few words on what else I would like to work on here at the University of Twente.

I hope to contribute to educating engineers that make humane technologies; technologies that are well embedded in society. To realize this, I have the ambition to give guest lectures in all the different honours programmes here at the UT: from Mathematics to Science, and from Philosophy to Processes of Change. My work is not only fundamental, curiosity driven research but also has the potential for practical applications. I would like to collaborate with engineers and scientists here on campus who are interested in developing ways of living better with technologies. Given global challenges such as climate change, screen-addiction, and obesity, I believe that the possibility of breaking our habits is urgent at this moment in time. However, changing behavior is also notoriously difficult. There is a huge gap between *knowing that*, for example, flying too much or sitting too much is problematic, and *actually changing* one’s habits in everyday life. But when engineers, artists and philosophers of embodied cognitive science join forces we might be able to create new affordances that actually support people in breaking their habits when they want to. I believe (Rietveld, 2016) that if we manage to radically change the affordances available in our surroundings, we will be able to generate behavioral change.

**Figure 23.**
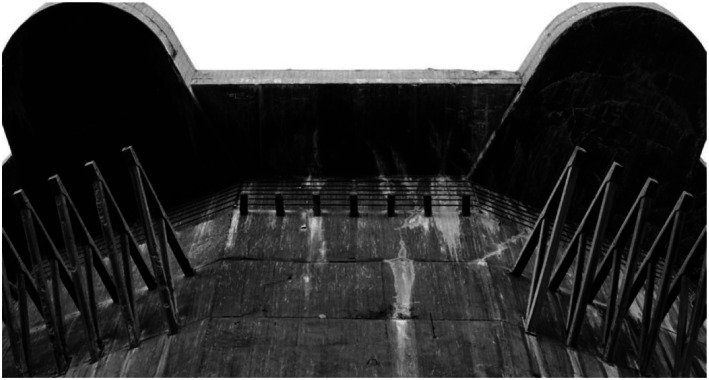
Still frame *Luftschloss*. RAAAF.

I would like to end this inaugural lecture by showing a final movie, *Luftschloss*, that brings together the three skills I have been discussing today. *Luftschloss* is a short movie by RAAAF on historically burdened heritage (Figure 23) that is transformed into an artwork. For making *Luftschloss* we used an interesting technology called hydro-demolition. Deconstruction occurs by using the force of water. A focused “water-lance” has the power and force to blast concrete into pieces. Only the reinforcement steel that is inside the concrete remains.

Using the *Luftschloss* project, I can wrap up what we have learned with respect to the three aspects of RAAAF’s work that I have been talking about: the skills of working with layers of meaning, creating material playgrounds, and an openness to the possibility of having radically different socio-material practices.

**Figure 24.**
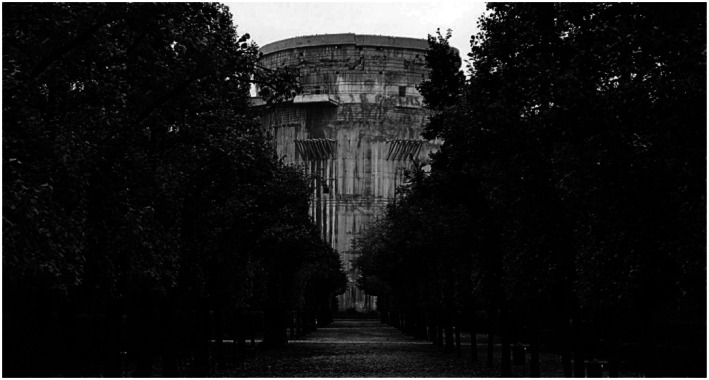
Flak tower, Vienna. Historically burdened heritage. Photo: Thomas Ledl.

**Figure 25.**
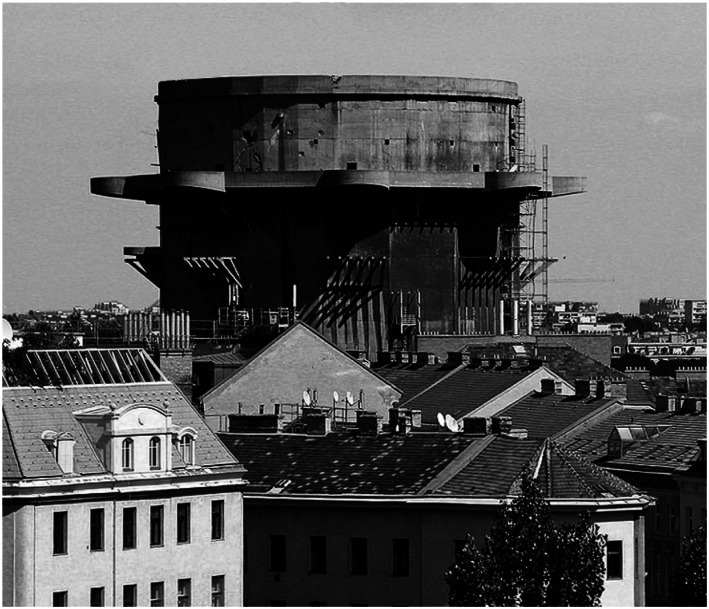
Flak tower in the center of Vienna. Still from *Luftschloss*

You will see in the *Luftschloss* movie that we made a big effort to create a site-specific, or better, situation-specific intervention. This is a way of *weaving multiple meanings* into the work. When we make situation-specific work at RAAAF, we try to be precise in dealing with the layered question: why this here now?^6^ The heritage object, a so-called “Flak Tower,” is a Nazi “castle” in Vienna and we basically strip away the original intention of it being a great monument of the regime that has built it (Figure 24 and 25).

In the movie, we transform the tower into an artwork in the center of Vienna and generate multiple layers of meaning with it. As such it becomes exemplary of how RAAAF’s art-based approach can deal meaningfully with historically burdened heritage, which is typically left untouched because no one wants to get their fingers burned when addressing it. Think of places related to slave trade here in The Netherlands, nuclear power plants, or places related to the Atlantic Wall and other European terrorscapes of the world wars. *Luftschloss* questions the current practice of leaving this kind of inhumane heritage untouched and suggests a way of transforming it into a site-specific artwork. *Luftschloss puts imagination central in our reflection* of how we could deal with heritage from a troubled past (see Rietveld & Rietveld, 2017). As an artwork, *Luftschloss* raises all sorts of new open questions, such as what does it mean that what remains of this fortress is a fragile, elegant, and unfolding skeleton?

**Figure 26.**
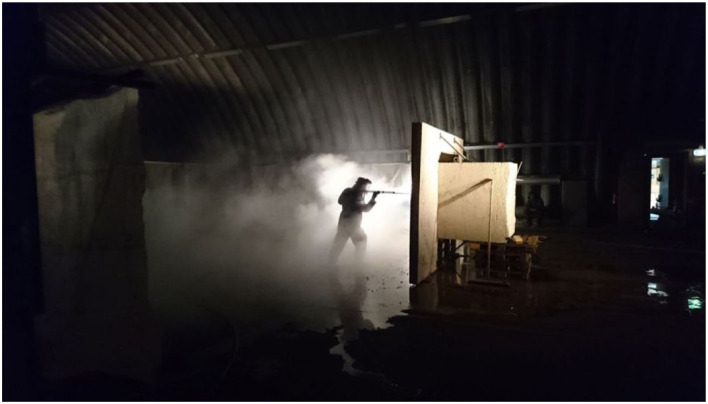
Material playground. Exploring the possibilities of hydro-demolition for *Luftschloss*. Photo: RAAAF.

**Figure 27.**
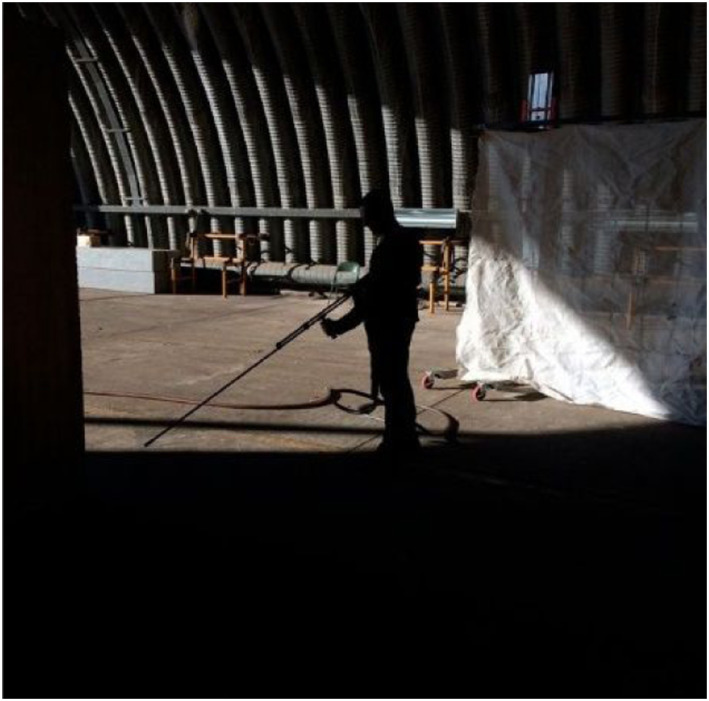
Material playground. Photo: RAAAF.

**Figure 28.**
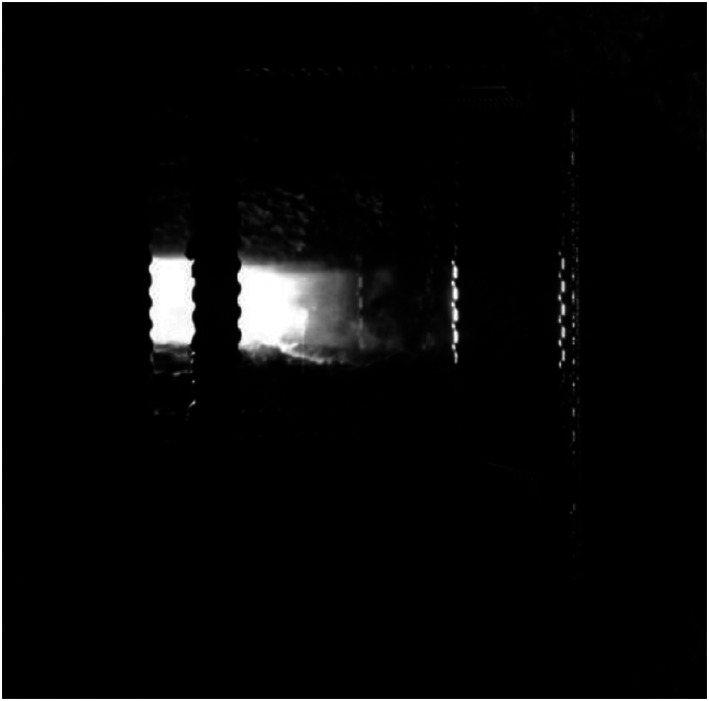
Reinforcement steel revealed. Still from *Luftschloss*.

The material playground we created in this project explored the possibilities of state-of-the art hydro-demolition technology (Figure 26). We used the focused, high pressure “water-lance” to destroy meters of concrete around the reinforcement steel (Figures 27 and 28). We started to work in this way out of our fascination with the power of water and by the enormous destructive force of this technology. We felt the urge to experience it ourselves and play with it. Making a material playground for that, we built a kind of set where we could test this technology and its power. This playground constructed for *Luftschloss* sets up conditions for testing and filming the process of demolishing reinforced concrete like that of the Flak Tower.

Moreover, the skills of architecture historians and architects allowed us to find secret original construction drawings, which we used to imagine and design this 3-D world. For animating this 3-D world we used the latest advances in digital technology. So, in this case we created not only a physical, material playground to explore and imagine the possibilities of a new technology, but also a digital one. The resulting movie^7^ afforded sharing with you our vision of how we could transform this kind of burdened heritage.

Third and finally, I would like to address the openness to the possibility of having radically different practices involved in *Luftschloss*. The artwork creates new combinations of technologies and new affordances. We try to open-up the imagination for the possibility of transforming an entire practice of cultural heritage. It is the current practice of governments and cultural heritage agencies to deal with historically burdened cultural heritage by looking away and leaving it untouched. The movie affords reflecting on how we could deal with this kind of heritage practice differently. One of the possibilities made concrete is showing how we could change the practice by setting an example in the form of an artwork. Finally, I hope the movie creates a widely shared *desire* for realizing this artwork there in Vienna.

Ik heb gezegd.

## Notes

1. This example is based on story told by Adriaan Geuze during an event at the Royal Netherlands Academy of Arts and Sciences, KNAW, December 10th, 2018.

2. See De Haan et al. (2013, 2015) for investigations of what it means for individuals to live with the medical technology of Deep Brain Stimulation.

3. To use the words of the late Lebbeus Woods: “[Show] what the world would be like if we were free from conventional limits … show what could happen if we lived by a different set of rules” (Woods in Ouroussof, 2008).

4. Erick de Lyon told me this story (personal communication June 10, 2019).

5. Thanks to Julian Kiverstein for pointing me to the BBC News article on this issue (Rodgers, 2018).

6. Thanks to our close collaborator Barbara Visser for putting it like this in conversation.

7. This movie is part of the collection of the Eye Film Museum Amsterdam, which is why there is no link included here.
